# Substrate-specific inhibition of organic cation transporter 1 revealed using a multisubstrate drug cocktail

**DOI:** 10.1016/j.dmd.2025.100074

**Published:** 2025-03-28

**Authors:** Vincent Rönnpagel, Felix Morof, Sarah Römer, Marleen J. Meyer-Tönnies, Mladen V. Tzvetkov

**Affiliations:** Department of General Pharmacology, Institute of Pharmacology, Center of Drug Absorption and Transport (C_DAT), University Medicine Greifswald, Greifswald, Germany

**Keywords:** OCT1, DDI, drug cocktail, substrate-specific effects

## Abstract

Transporters of the *SLC22* family, such as organic cation transporter 1 (OCT1), possess very broad substrate specificity. It is unclear to what extent the inhibitory potencies of OCT1 depend on the substrate used. Here, we describe a multisubstrate drug cocktail that allows for the simultaneous testing of drug-drug interactions using 8 different victim drugs: fenoterol, salbutamol, sumatriptan, zolmitriptan, ipratropium, trospium, methylnaltrexone, and metformin. There were no significant differences in Michaelis constant (K_M_) and v_max_ of the OCT1-mediated uptake of the substrates alone or in the cocktail. Depending on the victim drug analyzed, we observed 6.7-fold differences in the inhibitory potency of fenoterol (IC_50_ of 0.75 *μ*M for metformin and 5.1 *μ*M for sumatriptan). Similarly, the inhibitory potency of verapamil varied 6.7-fold (IC_50_ of 1.3 *μ*M for zolmitriptan and 8.7 *μ*M for ipratropium). Two groups of inhibitors showed strong correlations in their victim-dependent inhibitory potencies. Group 1 comprised verapamil, quinidine, fenoterol, and ipratropium, and group 2 comprised metformin, sumatriptan, and trimethoprim. By comparing OCT1 paralogs and orthologs, the broadest substrate spectra were observed for OCT1 and multidrug and toxin extrusion 1, followed by OCT2, multidrug and toxin extrusion 2-K, and OCT3. In contrast, organic cation transporters novel 1 and organic cation transporters novel 2 exhibited very narrow substrate specificity, transporting only L-carnitine and L-ergothioneine, respectively. In conclusion, OCT1 demonstrates substantial differences in inhibitory potencies, depending on the victim drug used. We developed a cocktail approach that enables rapid screening for such differences, facilitating the identification of drug-drug interactions at the early stages of drug development. This approach can be extended to other transporters with broad substrate specificity.

**Significance Statement:**

Polyspecific transporters have a broad substrate-binding cavity with no defined single binding position. Consequently, inhibitors may exhibit different inhibitory potencies depending on the victim drug used for testing. Here, we demonstrate this for organic cation transporter 1 (OCT1, SLC22A1) and presents a drug cocktail designed to identify varying inhibitory potencies in vitro and prevent false-negative drug-drug interaction results during early drug development. This approach can be extended to other polyspecific drug transporters.

## 1 Introduction

Transporters of the solute carrier family 22 (*SLC22*) are involved in the uptake and elimination of a variety of drugs, toxins, and endogenous compounds (Nigam, 2018). This is achieved by passive transport, either through facilitated diffusion (eg, for organic cation transporters [OCT] 1-3), through symport with sodium (eg, for organic zwitterion transporters organic cation transporters novel (OCTN) 1 and 2), or through antiport (eg, organic anion transporter OAT1 and uric acid transporter URAT1; Yee and Giacomini, 2021). OCT1 and OCT2 play a key role in the hepatic and renal clearance, respectively, and have a partially overlapping substrate spectrum. OCT2 is mainly expressed in the basolateral membrane of renal proximal tubules, where it mediates the secretion of drugs. OCT1 is predominantly expressed in the sinusoidal membrane of hepatocytes, where it mediates the first step in hepatic metabolism or excretion (Gorboulev et al, 1997; Zhang et al, 1997; Jonker et al, 2003; Koepsell et al, 2007).

OCTs have a broad substrate spectrum. OCT substrates show a highly variable molecular structure (Koepsell, 1998; Meyer and Tzvetkov, 2021). These include model cations such as TEA^+^, clinically relevant drugs such as metformin, fenoterol, sumatriptan, and ipratropium, as well as endogenous compounds such as thiamine (Shu et al, 2008; Nies and Schwab, 2010; Hendrickx et al, 2013; Chen et al, 2014, 2017; Matthaei et al, 2016; Tzvetkov et al, 2018). For some of the clinically relevant drugs, genetically determined loss or reduction of OCT1 function leads to a change in pharmacokinetics and liver concentrations (Shu et al, 2007; Tzvetkov et al, 2009, 2013; Sundelin et al, 2017; Tzvetkov et al, 2018).

In the context of preclinical drug development, OCT1 is slowly gaining more recognition and is recommended for testing of drug-drug interactions (DDIs) by the International Transporter Consortium (ITC; Zamek-Gliszczynski et al, 2018a,b; Galetin et al, 2024). According to the European Medicines Agency (EMA), testing for in vitro DDIs at OCT1 could be considered (EMA, 2015), whereas the US Food and Drug Administration (FDA) continues to recommend testing only for OCT2 (FDA, 2020).

Metformin, a biguanide that is used to treat type 2 diabetes, is a well-known OCT1 substrate. One suggested mechanism of metformin action is inhibiting gluconeogenesis in the liver (Foretz et al, 2014; Dutta et al, 2023; Corcoran and Jacobs, 2024), the organ of strongest OCT1 expression. However, OCT1-mediated metformin uptake into hepatocytes does not seem to be associated with metformin efficacy in humans (Zhou et al, 2009; Dujic et al, 2017). Metformin has been recommended as a substrate for testing DDIs at OCT1 in vitro (Shu et al, 2007) but not in vivo due to the missing effects of OCT1 on metformin pharmacokinetics and efficacy in humans (Zhou et al, 2009; Dujic et al, 2017; Galetin et al, 2024; Paglialunga et al, 2024).

Fenoterol is a beta-2 adrenergic receptor agonist that is used to treat asthma or chronic obstructive pulmonary disease. Individuals homozygous for OCT1 loss-of-function genetic variants had a 2-fold increase in systemic fenoterol exposure and an increased risk for cardiovascular and metabolic adverse reactions (Tzvetkov et al, 2018).

Sumatriptan is a 5-HT_1_ receptor agonist that is used to treat migraine. Individuals homozygous for OCT1 loss-of-function genetic variants had a 2-fold increase in systemic sumatriptan exposure (Matthaei et al, 2016). Fenoterol and sumatriptan may be suitable substrates for testing DDIs at OCT1 in vivo because the loss of OCT1 function by inhibition or genetic variants affects their pharmacokinetics in vivo.

No model OCT1 inhibitor for in vivo use has been recommended by the EMA, ITC, or International Council for Harmonisation of Technical Requirements for Pharmaceuticals for Human Use (ICH) yet. However, recently, sumatriptan was used successfully as a victim drug to reveal DDIs at OCT1 in vivo (Wang et al, 2023). Several studies have also identified potential OCT1 inhibitors, including trimethoprim (an antibiotic drug; Jung et al, 2008; Vora et al, 2023), verapamil (a calcium antagonist; Morse et al, 2020; Koepsell, 2021), and quinidine (an antiarrhythmic drug; Shu et al, 2007; Zhou et al, 2021). These 3 inhibitors have similar inhibitory potencies for metformin uptake via OCT1, ie, 7.7 *μ*M for quinidine, 9.6 *μ*M for trimethoprim, and 12.5 *μ*M for verapamil (Ahlin et al, 2011; Panfen et al, 2019).

A cocktail approach is well established for analyzing multiple transporters in a single administration in humans (Paglialunga et al, 2024). Here, we hypothesize that using a cocktail in vitro may be also helpful in identifying substrate-specific DDIs in the case of OCT1 as an example of a transporter with broad substrate specificity. The aim of this study was to establish a cocktail of OCT1 substrates and use it to assess DDIs with multiple victim drugs in vitro.

## 2 Materials and methods

### 2.1 Reagents

Ammonium chloride (NH_4_Cl), fenoterol hydrobromide, salbutamol hydrochloride, sumatriptan succinate, zolmitriptan, methylnaltrexone bromide, metformin hydrochloride, L-carnitin-d9, L-ergothioneine, verapamil hydrochloride, trimethoprim, and fenoterol-d6 were obtained from Sigma-Aldrich; ipratropium bromide, trospium chloride, sumatriptan-d6, and isobutyryl-L-carnitine-d6 chloride were obtained from Santa Cruz Biotechnology; trospium-d8 was obtained from Toronto Research Chemicals; and quinidine sulfate dihydrate and dimethyl sulfoxide were obtained from Carl Roth. All chemicals that were used in this study had purities greater than 97%. Dulbecco’s Modified Eagle’s Medium, Hanks’ Buffered Salt Solution (HBSS), fetal bovine serum, and Pierce BCA Protein Assay were obtained from Thermo Fisher Scientific. Penicillin-streptomycin was obtained from PAN-Biotech. HEPES was obtained from Carl Roth, and poly-D-lysine (1–5 kDa) hydrobromide was obtained from Sigma-Aldrich. Twelve-well plates were obtained from Starlab, and tissue-culture flasks were from Sarstedt. Acetonitrile, methanol, and formic acid in liquid chromatography-tandem mass spectrometry (LC-MS/MS) grade were obtained from Merck.

### 2.2 Cell lines and cell culturing

In this study, human embryonic kidney (HEK) 293 cells stably overexpressing mouse Oct1 (mOct1), human OCT1 (hOCT1), human OCT2 (hOCT2), human OCT3 (hOCT3), human OCTN1 (hOCTN1), human OCTN2 (hOCTN2), human OAT1 (hOAT1), human OAT3 (hOAT3), human multidrug and toxin extrusion (MATE)1 (hMATE1), and human MATE2K (hMATE2K) were used. The generation of cell lines by targeted chromosomal integration has been described previously (Tzvetkov et al, 2012; Seitz et al, 2015). hOCT3 was a kind gift from Jürgen Brockmöller and Kyra Redeker (Institute of Clinical Pharmacology, University Medicine Göttingen). Cells were cultured with Dulbecco’s modified Eagle’s medium supplemented with 10% FBS, 100 U/mL penicillin, and 100 *μ*g/mL streptomycin, and the cells were maintained at 37 °C and 5% CO_2_.

### 2.3 Cellular uptake and inhibition experiments

Before seeding cells, 12-well plates were precoated with a solution of 2 mg/mL poly-D-lysine. Cells were seeded at a density of 6 × 10^5^ cells/mL/well and cultivated for 48 hours before initiating experiments. All experiments were performed at 37 °C and pH 7.4 using HBSS+ (HBSS supplemented with 10 mM HEPES). First, the cells were washed with 1 mL of HBSS+. For hMATE1 and hMATE2K, the cells were additionally preincubated with 1 mL of HBSS++ (HBSS+ additionally supplemented with 30 mM NH_4_Cl) for 30 minutes. In both cases, the uptake or inhibition experiments were initiated by replacing HBSS+/HBSS++ with 400 *μ*L of prewarmed HBSS+ containing the substrates or a mix of substrate and inhibitor, respectively. Uptake was stopped for hMATE1 and hMATE2K after 1 minute and for the rest after 2 minutes by adding 2 mL of ice-cold HBSS+. Every well was washed twice with 2 mL ice-cold HBSS+, and the cells were lysed in 500 *μ*L of 80% acetonitrile containing the internal standards (IS, Table 1). Intracellular substrate concentrations were measured by LC-MS/MS and normalized to the total amount of protein as measured using the bicinchoninic acid assay (Smith et al, 1985).

**Table 1:** Parameters for the quantitative liquid chromatography-tandem mass spectrometry detection of the cocktail substrates

Analyte	MRM (m/z)	CE [Volt]	RT [Min]	Internal Standard	IS MRM (m/z)	CE [Volt]	RT [Min]
Fenoterol	304.3/107.1	50	5.45	Fenoterol-d6	310.3/141.2	25	5.42
Salbutamol	240.3/148.1	25	3.88				
Sumatriptan	296.2/58.1	43	5.03	Sumatriptan-d6	302.4/64.2	45	5.02
Zolmitriptan	288.5/58.1	50	5.37				
Ipratropium	332.3/166.3	35	6.32				
Methylnaltrexone	356.4/284.2	35	4.91				
Metformin	129.9/71	32	1.68				
Trospium	392.3/164.3	43	7.65	Trospium-d8	401.4/172.3	44	7.63
L-carnitine-d9	171.2/103.2	26	1.61	Isobutyryl-L-carnitine-d6	238.40/58.1	27	3.64
L-ergothioneine	230.3/127.2	27	2.02				

CE, collision energy; MRM, multiple reaction monitoring; RT, retention time.

### 2.4 Quantification of intracellular substrate concentration by LC-MS/MS

For sample preparation prior to LC-MS/MS measurement, the cell debris was first removed by centrifugation at 16,000 × *g* for 15 minutes. A total volume of 350 *μ*L from each of the resulting supernatants was evaporated to dryness under nitrogen flow at 40 °C. The evaporated samples were then reconstituted in 200 *μ*L of 0.1% formic acid, and 10 *μ*L was injected into the LC-MS/MS system.

LC-MS/MS analysis was performed using an LC-30AD binary pump, a SIL-30AC autosampler, and a CTO-20AC column oven (Shimadzu Corporation) connected to a Sciex QTRAP 4000 triple quadrupole mass spectrometer (AB SCIEX). Electrospray ionization was used for MS detection in positive ion mode. Source parameters were set as follows: source temperature, 400 °C; ion spray voltage, 5 kV; collision gas, medium; curtain gas, 35 psi; ion source gas 1 and 2, 50 psi; dwell time, 50 ms; declustering potential, 50 V; entrance potential, 10 V; and collision cell exit potential, 10 V. Collision energy was optimized for each substrate individually (Table 1). Analyst 1.7.1 software (AB SCIEX) was used for data analysis.

Chromatographic separation of the cocktail substances was performed using a Brownlee SPP RP-Amide column (4.6 × 100 mm, 2.7 *μ*m; PerkinElmer). Solvent A consisted of 90% acetonitrile + methanol (6+1) and 10% 0.1% formic acid in water. Solvent B consisted of 0.1% formic acid in water. Separation was performed at a flow rate of 0.55 mL × min^−1^ using the following conditions: 0–5 minutes: 0% to 20% A; 5–7 minutes: 20% to 80% A; 7–7.01 minutes: 80% to 100% A; and 7–9.5 minutes: 0% A. The column temperature was set to 40 °C, and the autosampler temperature was 5 °C.

The preparation of stock, standard, and quality control samples is described in the Supplemental Materials, under the Supplemental Methods section. The validation of the LC-MS/MS method is detailed in the Supplemental Materials, under the Supplemental Results section.

### 2.5 Data analysis

The kinetic transport parameters K_M_ and v_max_ as well as the half-maximal inhibitory concentration (IC_50_) were calculated using GraphPad Prism version 8.0.2 (GraphPad Software, Inc). K_M_ and v_max_ were calculated using nonlinear regression to the Michaelis-Menten equation. IC_50_ values were calculated using nonlinear regression to the log(inhibitor) versus normalized response–variable slope with the following equation:

$$y=\frac{100}{1+10^{\left(\left(\log\;{IC}_{50}-x\right)\times hillslope\;\right)}\;}$$

Kinetic parameters from experiments using single substrates and those using the cocktail were compared using the Mann-Whitney U test. All inhibition experiments were analyzed using the Student’s *t* test with Bonferroni correction for multiple testing. Both statistical tests were performed using SPSS version 29 (IBM SPSS Statistics).

The risk of DDIs was assessed based on ITC recommendations to extend the existing FDA guidelines for hepatic transporters to OCT1 (FDA 2017; Zamek-Gliszczynski et al, 2018b). Therefore, the calculation method used for hepatic uptake transporters such as organic anion transporting polypeptide (OATP) 1B1 and OATP1B3 was also applied for OCT1. The DDI risk (R) as the ratio of the victim drugs’ area under the curve (AUC) in the presence or absence of inhibitor (perpetrator) was calculated as follows:

$$R=1+\frac{{fu}_{p}\times I_{in,\max}}{{IC}_{50}}$$

With fu_p_ as the fraction of drug that is unbound in plasma, IC_50_ the half-maximal inhibitory concentration, and I_in,max_ as the maximum concentration of inhibitor at the inlet of the liver. The R value reflects the estimated change in AUC, ie, a R value of 1.1 reflects a predicted change in AUC of 10%. In accordance to the current FDA guidelines (FDA, 2020), R ≥ 1.1 was used as cutoff value for estimated potential to inhibit OCT1 in vivo.

I_in,max_ was calculated as follows, and parameters used for calculation are listed in Supplemental Table 1.

$$I_{\text{in},\max}=I_{\max}+\frac{{F_{a}\times k}_{a}\times D}{Q_{h}}$$

With I_max_ as the maximum plasma concentration of inhibitor, F_a_ as the fraction absorbed after oral administration, k_a_ as the first-order absorption rate constant, D as the dose of inhibitor administered, and Q_h_ as hepatic blood flow. For fenoterol and ipratropium that are not administered orally, I_max_ was used as equal to I_in,max_. Pharmacokinetic parameters were obtained from Goodman & Gilman's (Hardman, 2018), except for fenoterol (Tzvetkov et al, 2018) and ipratropium (Zhang et al, 2023), and if not given, f_u_ was obtained from Lombardo et al (2018). K_a_ was calculated manually based on available pharmacokinetic parameters. Q_h_ was set to 97 L/h and F_a_ was assumed as 1.

## 3 Results

### 3.1 Development of a cocktail of OCT1 substrates and a method for their simultaneous quantification

To develop a multisubstrate cocktail, we chose substrates that are (1) clinically relevant, (2) strongly transported by OCT1 in vitro, and (3) represent the high structural diversity of OCT1 substrates. The cocktail contains 8 drugs. Five of them are commonly used drugs that represent different substrate structures, ie, metformin, sumatriptan, fenoterol, trospium, and methylnaltrexone. Additionally, we included zolmitriptan, salbutamol, and ipratropium, which share structural similarities with sumatriptan, fenoterol, and trospium, respectively. This allowed for the simultaneous analysis of the effects of both major and minor structural changes.

The substrate concentrations in the cocktail were selected to be far below their K_M_ values for hOCT1 and mOct1 (Table 2) to minimize the risk of DDIs within the cocktail.

**Table 2:** Substrate concentrations in the cocktail and their K_M_ values for hOCT1 and mOct1

Substrate	Concentration Used in the Cocktail [*μ*M]	K_M_ hOCT1 [*μ*M]	K_M_ mOct1 [*μ*M]	References
Fenoterol	0.5	1.8	6.9	(Tzvetkov et al, 2018; Morse et al, 2020; Jensen et al, 2020; Meyer et al, 2022)
Salbutamol	5	310	52.2	(Jensen et al, 2020; Meyer et al, 2022)
Sumatriptan	0.5	55.8	65.7	(Tzvetkov et al, 2018; Morse et al, 2020; Meyer et al, 2022)
Zolmitriptan	0.5	105	n/a	(Wittern et al, 2024), own unpublished data
Ipratropium	0.5	12.2	6.06	(Tzvetkov et al, 2018; Meyer et al, 2022)
Trospium	0.1	16.1	1.81	(Tzvetkov et al, 2018; Meyer et al, 2022)
Methylnaltrexone	1	15.2	8.61	(Meyer et al, 2019; Meyer et al, 2022)
Metformin	100	3030	491	(Elsby et al, 2017; Tzvetkov et al, 2018; Meyer et al, 2022)

K_m_, Michaelis constant; OCT, organic cation transporter; n/a, not available.

**Fig. 1:**
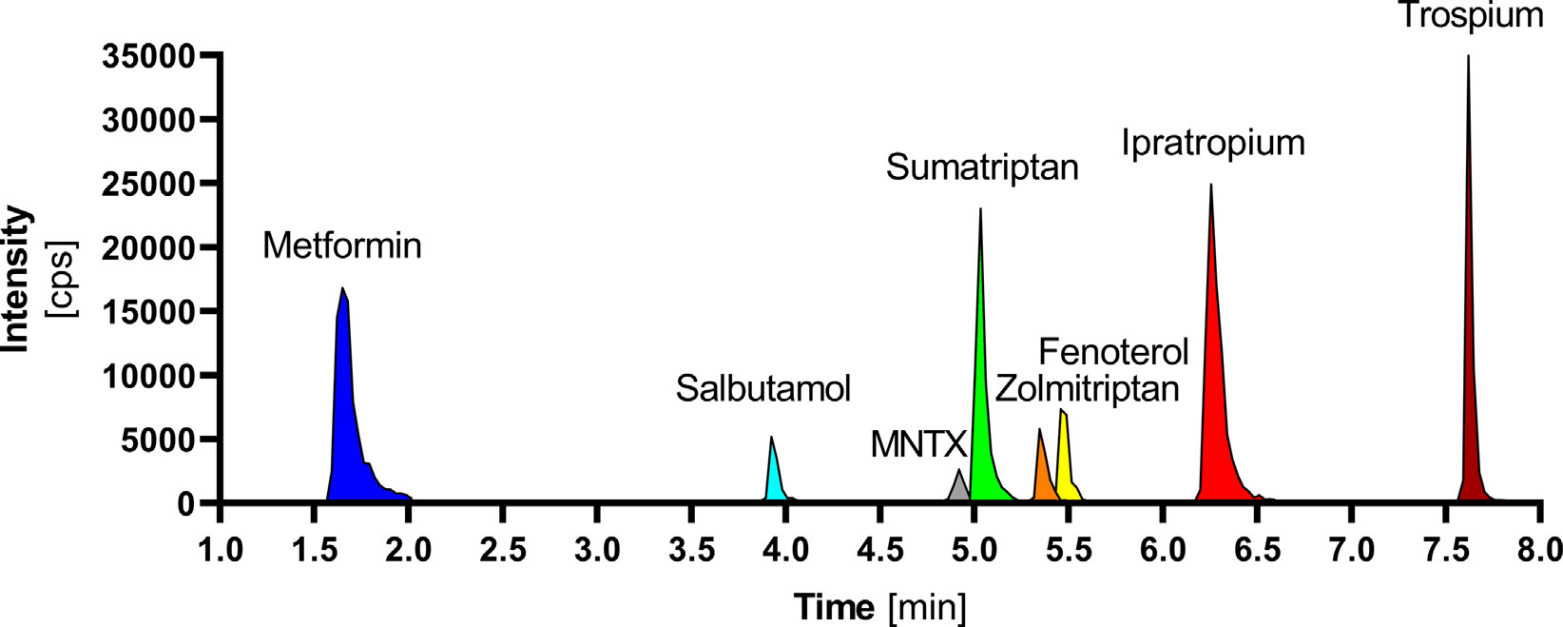
The analytical method for simultaneous quantification of the OCT1 cocktail substrates. Chromatogram of the cocktail substrates showing the substrate-selective peaks labeled in different colors (metformin dark blue, salbutamol light blue, methylnaltrexone gray, sumatriptan green, zolmitriptan orange, fenoterol yellow, ipratropium red, and trospium dark red). Separation conditions and MS/MS parameter are described in the methods section.

We developed an LC-MS/MS method that allowed the simultaneous detection of all 8 cocktail substrates (Fig. 1) and followed the ICH guideline M10 on bioanalytical method validation and study sample analysis (ICH, 2023). The validation of the method is described in detail in the Supplemental Materials (Supplemental Tables 2–4).

### 3.2 Validation of the cocktail

**Fig. 2:**
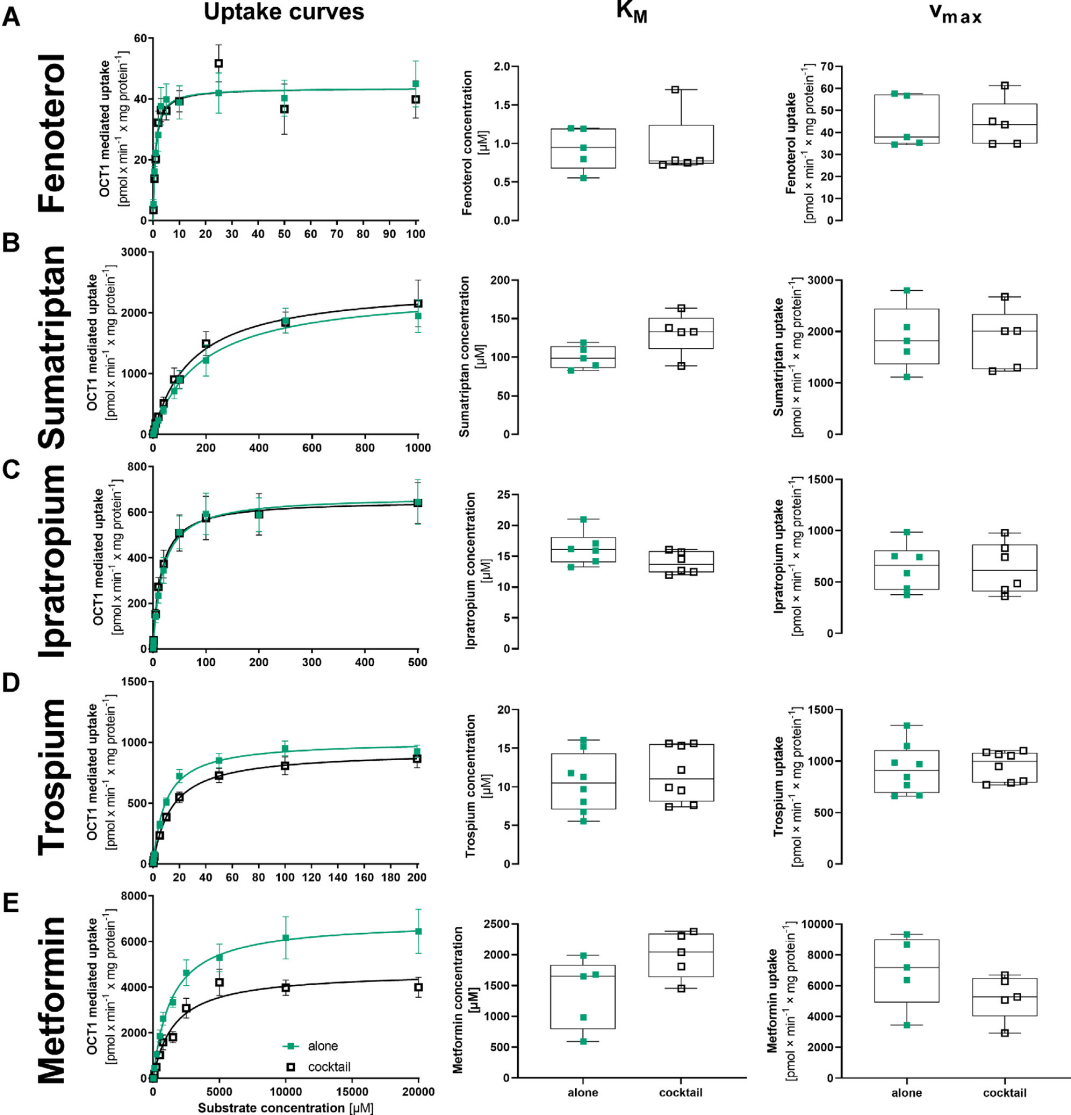
Comparison of the transport kinetics between measurement of substrate alone and in the cocktail for fenoterol (A), sumatriptan (B), ipratropium (C), trospium (D), and metformin (D). HEK293 cells stably overexpressing human OCT1 were incubated with increasing concentrations of the substrates either alone (green boxes) or as a cocktail (black open boxes). OCT1-mediated uptake was calculated by subtracting the uptake of the empty vector control. Shown are the concentration-dependent uptake curves as means and standard errors of the means (left panel), and absolute values for K_M_ (middle panel) and v_max_ (right panel) for single experiments and as median and quantiles. *n* = 5–8 independent experiments.

To validate the cocktail, we analyzed whether the combination of all substrates in a cocktail affected their individual transport kinetics in terms of K_M_ and v_max._ We measured the uptake using HEK293 cells stably overexpressing human OCT1. To measure the transport kinetics for a single substrate in the cocktail, we increased the concentration of 1 substrate while keeping the concentrations of the other substrates in the cocktail constant. The resulting K_M_ and v_max_ values were compared with those obtained when each substrate was tested alone (Fig. 2; Supplemental Table 5).

We validated 5 representative substrates from the cocktail. For fenoterol, ipratropium, trospium, and sumatriptan, no differences in K_M_ or v_max_ were observed when measured alone and in the cocktail (Fig. 2, A–D). For metformin, a trend toward lower v_max_ and higher K_M_ in the cocktail compared with measurement alone was observed, but the difference was not statistically significant (*P* = .095 and .1508 for K_M_ and v_max_, respectively; Fig. 2E). Based on this, we conclude that the multisubstrate cocktail may be representative of the effects of each of the substrates alone.

### 3.3 Using the cocktail for assessing substrate-specific differences in inhibitory potencies

**Fig. 3:**
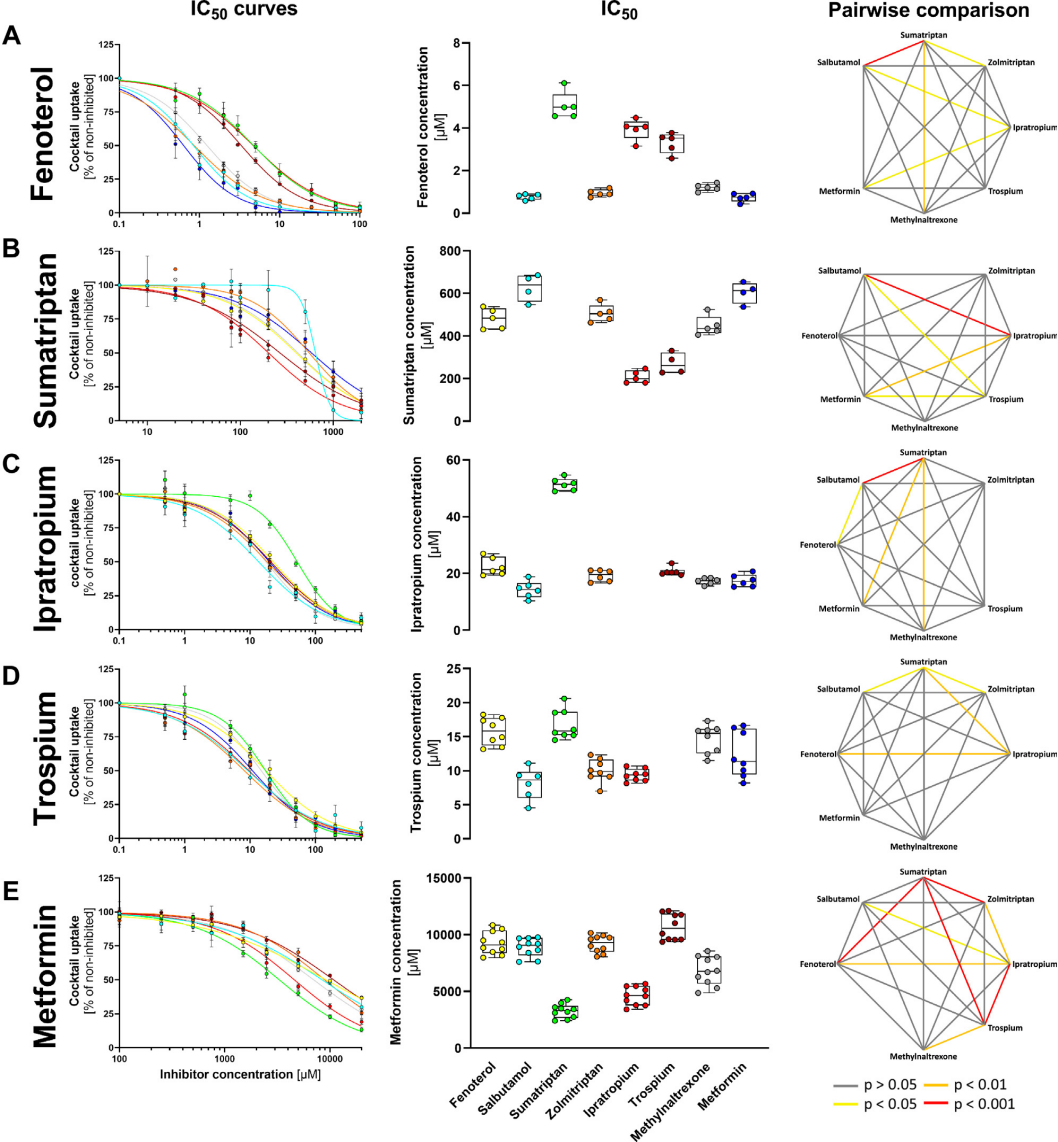
Comparison of substrate-specific differences in OCT1 inhibitory potencies of fenoterol (A), sumatriptan (B), ipratropium (C), trospium (D), and metformin (E). HEK293 cells were incubated with increasing concentrations of selected substrates while the concentration of the rest of the cocktail substrates remained constant. OCT1-mediated uptake was calculated by subtracting the uptake of the empty vector control. Shown are inhibition curves as means and standard errors of the means (left panel) and the IC_50_ values for single experiments as median and quantiles (middle panel). The respective substrates in the cocktail have the following colors: fenoterol yellow, salbutamol light blue, sumatriptan green, zolmitriptan orange, ipratropium red, trospium dark red, methylnaltrexone gray, and metformin dark blue. Additionally, the pairwise comparison of the differences in the inhibition potency as IC_50_ values between the different victim drugs is shown (right panel). Significance was calculated using the Kruskal–Wallis test with Dunn-Bonferroni post hoc-test. *n* = 5–10 independent experiments.

Next, we analyzed the inhibitory potencies of the OCT1 substrates included in the cocktail itself. To this end, we performed inhibition measurements by increasing the concentration of one of the substrates in the cocktail while keeping the concentrations of the others constant. We calculated the half-maximal inhibitory concentration IC_50_ and compared it between the substrates (Fig. 3).

As expected, the range of the inhibitory potencies reflected the affinity for OCT1 of each of the substrates/inhibitors used. The inhibitory potency in terms of IC_50_ of the high-affinity OCT1 substrate fenoterol varied between 0.7 and 5.1 *μ*M (Fig. 3A). The IC_50_ values of the low-affinity substrate metformin varied between 3.3 and 10.7 mM (Fig. 3E).

More interestingly, the observed IC_50_ values for some inhibitors were strongly dependent on the substrate used. Fenoterol inhibited the uptake of salbutamol, zolmitriptan, methylnaltrexone, and metformin with an on average 4.4-fold lower IC_50_ than it inhibited the uptake of trospium, ipratropium, and sumatriptan (Fig. 3A). In contrast, sumatriptan itself was a potent inhibitor of trospium and ipratropium (IC_50_ of 270 and 207 *μ*M, respectively; Fig. 3B), but a less potent inhibitor of fenoterol and metformin (IC_50_ of 482 and 604 *μ*M, respectively; Fig. 3B).

Next, we analyzed the inhibitory potencies of the known OCT1 inhibitors trimethoprim, quinidine, and verapamil on the uptake of the cocktail.

**Fig. 4:**
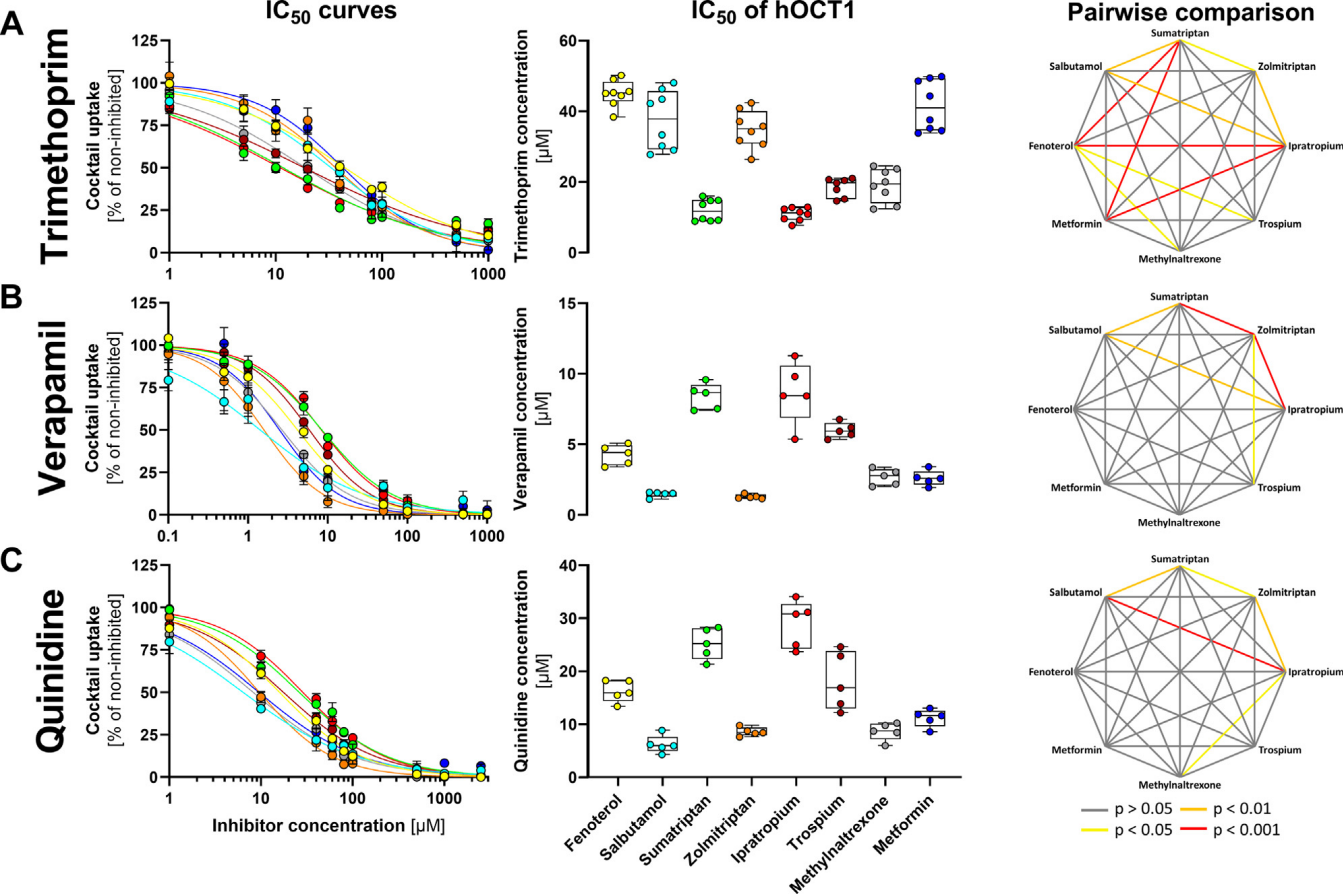
Inhibition of the cocktail with trimethoprim (A), verapamil (B), and quinidine (C). HEK293 cells were incubated with increasing concentrations of selected OCT1 inhibitors. OCT1-mediated uptake was calculated by subtracting the uptake of the empty vector control. Shown are inhibition curves as means and standard errors of the means (left panel) and the IC_50_ values for single experiments as median and quantiles (middle panel). The respective substrates in the cocktail have the following colors: fenoterol yellow, salbutamol light blue, sumatriptan green, zolmitriptan orange, ipratropium red, trospium dark red, methylnaltrexone gray, and metformin dark blue. Additionally, the pairwise comparison of the differences in the inhibition potency as IC_50_ values between the different victim drugs is shown (right panel). Significance was calculated using the Kruskal-Wallis-Test with Dunn-Bonferroni post hoc-test. n = 5–8 independent experiments.

Verapamil and quinidine showed similar substrate-specific effects that were distinct from trimethoprim. The inhibitory potency in terms of IC_50_ of verapamil and quinidine varied between 1.3 and 8.7 *μ*M (Fig. 4B) and 6.2 and 28.9 *μ*M (Fig. 4C), respectively. The IC_50_ values of trimethoprim varied between 10.9 and 45.1 *μ*M (Fig. 4A).

The inhibitory potencies for all 3 inhibitors were substrate-dependent. Both verapamil and quinidine inhibited the uptake of salbutamol, zolmitriptan, methylnaltrexone, and metformin with an on average 2.9-fold lower IC_50_ than they inhibited the uptake of sumatriptan, ipratropium, and trospium (Fig. 4, B and C). In contrast, trimethoprim inhibited sumatriptan, ipratropium, trospium, and methylnaltrexone with an on average 2.6-fold lower IC_50_ than it inhibited the uptake of fenoterol, salbutamol, zolmitriptan, and metformin (Fig. 4A). Only methylnaltrexone was inhibited with high potency by all 3 inhibitors tested.

Interestingly, structurally similar substrates of the same drug class showed different inhibition profiles. Zolmitriptan was inhibited 2.9-fold more potently than sumatriptan by quinidine and 6.5-fold by verapamil, but sumatriptan was inhibited 2.9-fold more potently than zolmitriptan by trimethoprim. Similar differences were observed for the potency of inhibition of trospium and ipratropium uptake. Salbutamol was inhibited more potently than fenoterol by all 3 inhibitors (Fig. 4 and Table 3).

**Table 3:** Half-maximal inhibitory concentration (IC_50_ in *μ*M; mean ± SD) for each cocktail substrate using different inhibitors

		Inhibitors							
		Fenoterol	Sumatriptan	Ipratropium	Trospium	Metformin	Trimethoprim	Verapamil	Quinidine
Substrates	Fenoterol		482 ± 42.9	22.4 ± 2.84	15.8 ± 1.87	9270 ± 954	45.0 ± 3.45	4.26 ± 0.63	16.3 ± 1.83
	Salbutamol	0.78 ± 0.12	628 ± 54.8	14.3 ± 2.66	8.14 ± 2.12	8882 ± 760	37.5 ± 7.77	1.48 ± 0.18	6.22 ± 1.48
	Sumatriptan	5.05 ± 0.57		51.4 ± 1.95	16.8 ± 2.03	3277 ± 575	12.0 ± 2.88	8.40 ± 0.82	25.2 ± 2.62
	Zolmitriptan	0.95 ± 0.15	506 ± 36.2	19.2 ± 1.82	10.0 ± 1.59	9192 ± 706	34.9 ± 4.97	1.30 ± 0.13	8.61 ± 0.71
	Ipratropium	3.95 ± 0.44	207 ± 26.1		9.37 ± 0.83	4621 ± 766	10.8 ± 1.76	8.66 ± 1.95	28.9 ± 3.95
	Trospium	3.31 ± 0.43	270 ± 42.6	20.6 ± 1.35		10,685 ± 1093	18.5 ± 2.48	5.99 ± 0.48	18.1 ± 4.88
	Methylnaltrexone	1.21 ± 0.15	448 ± 40.9	17.3 ± 1.01	14.7 ± 1.95	6834 ± 1203	19.0 ± 4.36	2.69 ± 0.52	8.67 ± 1.48
	Metformin	0.75 ± 0.18	604 ± 42.3	17.4 ± 1.95	12.4 ± 3.14		41.7 ± 6.65	2.61 ± 0.48	11.2 ± 1.48
Minimum		0.75 ± 0.18	207 ± 26.1	14.3 ± 2.66	8.14 ± 2.12	3277 ± 575	10.8 ± 1.76	1.30 ± 0.13	6.22 ± 1.48
Maximum		5.05 ± 0.57	628 ± 54.7	51.4 ± 1.95	16.8 ± 2.03	10,685 ± 1093	45.0 ± 3.45	8.66 ± 1.95	28.9 ± 3.95
Range		6.7	3.0	3.6	2.1	3.3	4.2	6.7	4.6

**Fig. 5:**
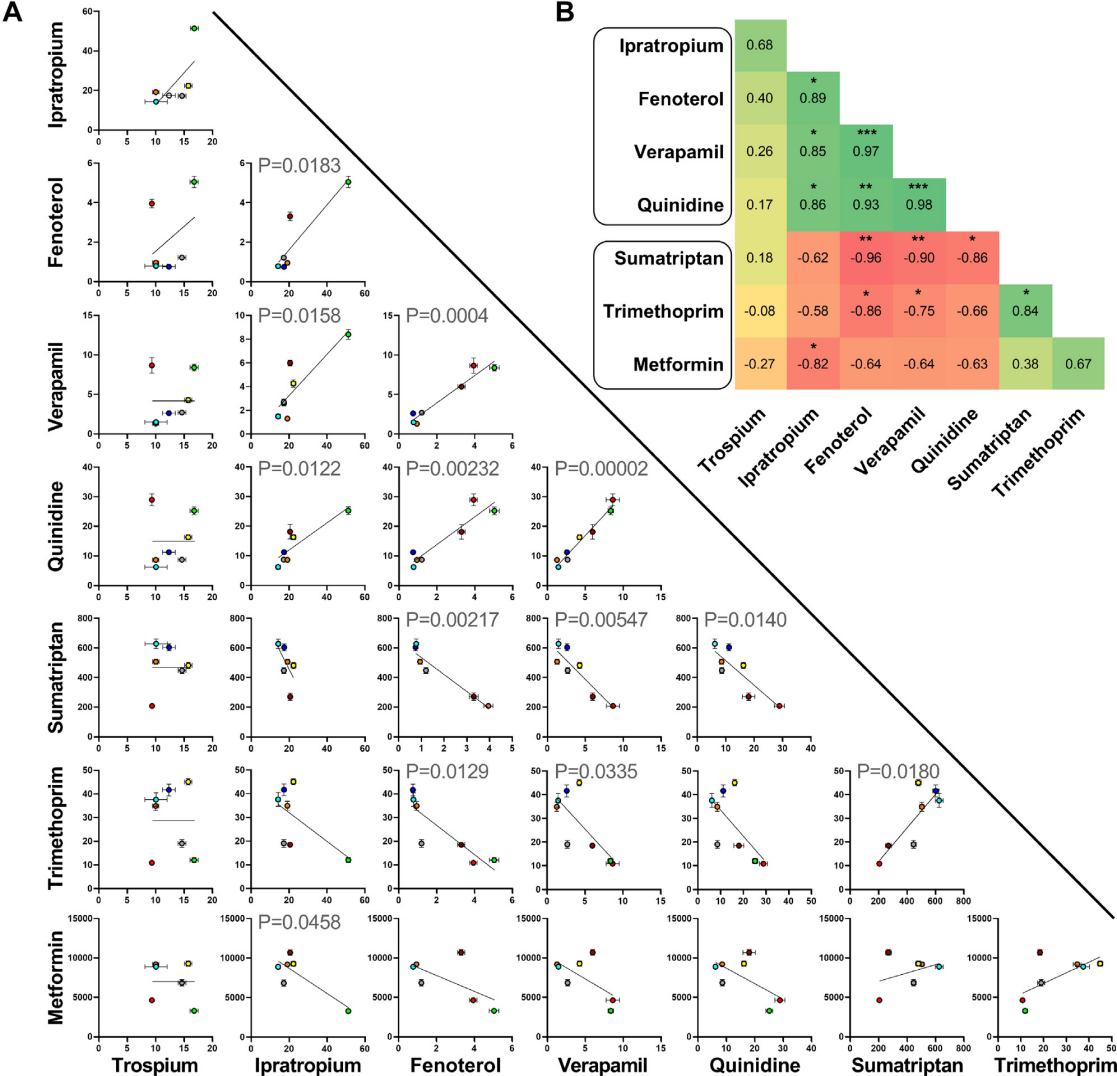
Correlation of the inhibitory potencies of different inhibitors on the cocktail uptake via OCT1. Shown is the pairwise correlation as individual graphs (A) and the Pearson correlation coefficients in a heat map (B). Red indicates negative correlation, green indicates positive correlation, and yellow represents no correlation. *n* = 6–8 independent experiments ∗ *P* < .05, ∗∗ *P* < .01, ∗∗∗ *P* < .001.

Two distinct groups of inhibitors were identified that showed significant positive correlations in their inhibitory potencies within the groups and a negative correlation between the 2 groups (Fig. 5). The first group included fenoterol, quinidine, verapamil, and partially ipratropium, whereas the second group consisted of sumatriptan, trimethoprim, and partially metformin. Interestingly, trospium showed neither a positive nor a negative correlation with any of the other inhibitors tested.

### 3.4 Using the cocktail to assess substrate selectivity of OCT1 paralogs

We used the established cocktail to analyze the substrate selectivity of the human OCT1 paralogs OCT2, OCT3, OCTN1, OCTN2, OAT1, and OAT3, as well as transporters of organic cations from other families—MATE1 and MATE2K (*SLC47A1* and *SLC47A2*, respectively). To this end, the cocktail was expanded by L-ergothioneine (1 *μ*M) and L-carnitin-d6 (1 *μ*M), which are known substrates of OCTN1 and OCTN2.

**Fig. 6:**
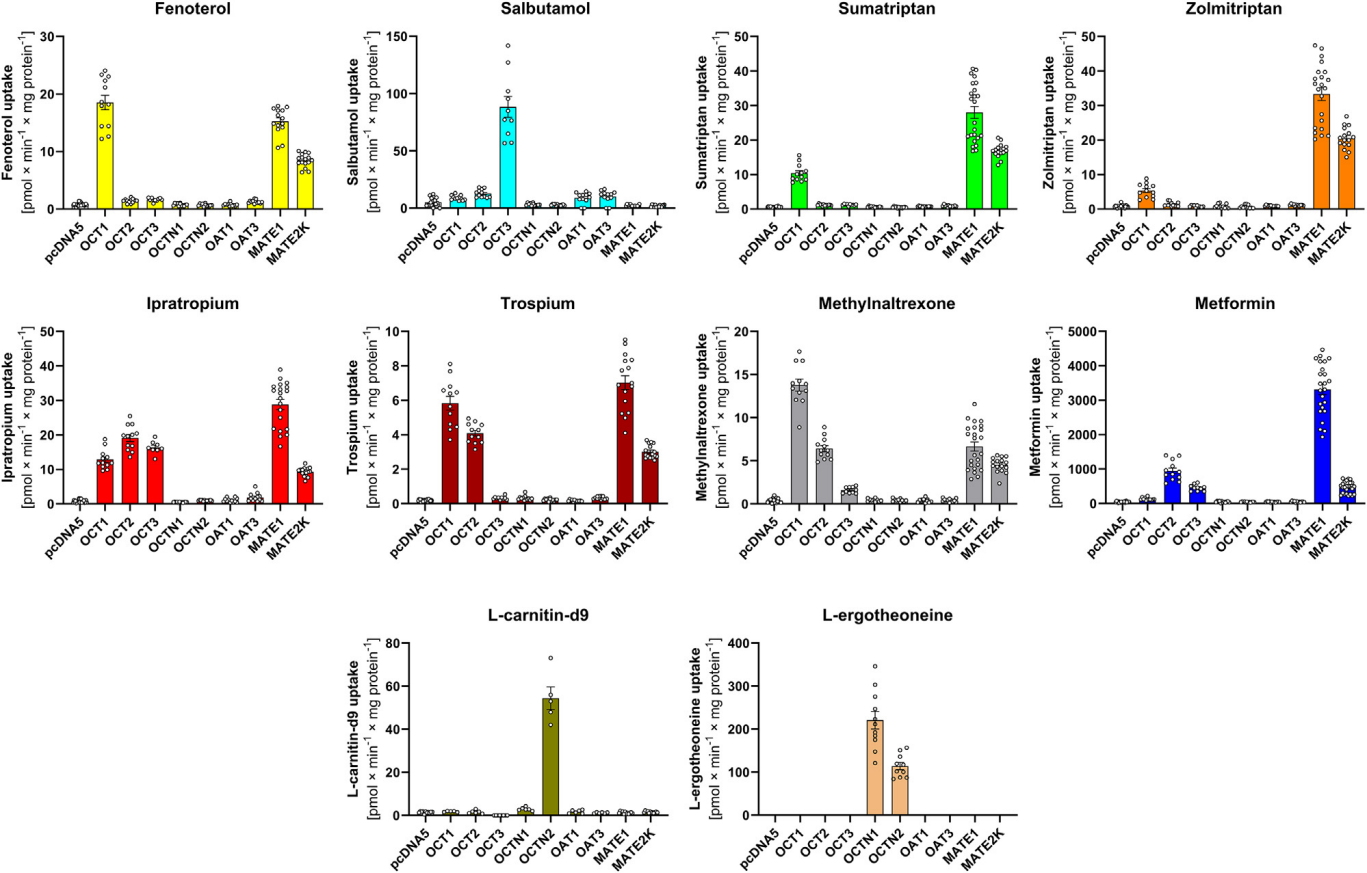
Assessing differences in the uptake between hOCT1 and its paralogs. HEK293 cells were incubated with the cocktail extended with L-carnitin-d6 and L-ergothioneine to compare the effect of extending the cocktail and to look at the transport of the different isoforms. The transporter-mediated uptake was calculated by subtracting the uptake of the empty vector control. Shown are means ± SEM; *n* = 12 independent experiments.

We observed strong differences in substrate selectivity even between closely related transporters such as OCT1, OCT2, and OCT3 (Fig. 6). Although fenoterol was transported only by OCT1, the structurally related salbutamol was transported only by OCT3. Similarly, ipratropium was transported by all 3 OCTs, but the highly structurally related trospium was transported only by OCT1 and OCT2.

In contrast to the OCTs, the OCTNs showed a very narrow substrate spectrum. OCTN2 transported only L-carnitine, and OCTN1 transported both L-carnitine and L-ergothioneine, but none of the remaining substrates in the cocktail (Fig. 6). As expected, OAT1 and OAT3 did not transport any of the substrates analyzed.

MATEs exhibited the broadest substrate specificity among all transporters tested. They transported all substrates in the basic cocktail except for salbutamol. For some substrates, such as metformin and sumatriptan, MATEs possessed by far the strongest transport activity.

### 3.5 Comparison of the substrate-specific differences in inhibitory potencies between human and mouse OCT1

**Fig. 7:**
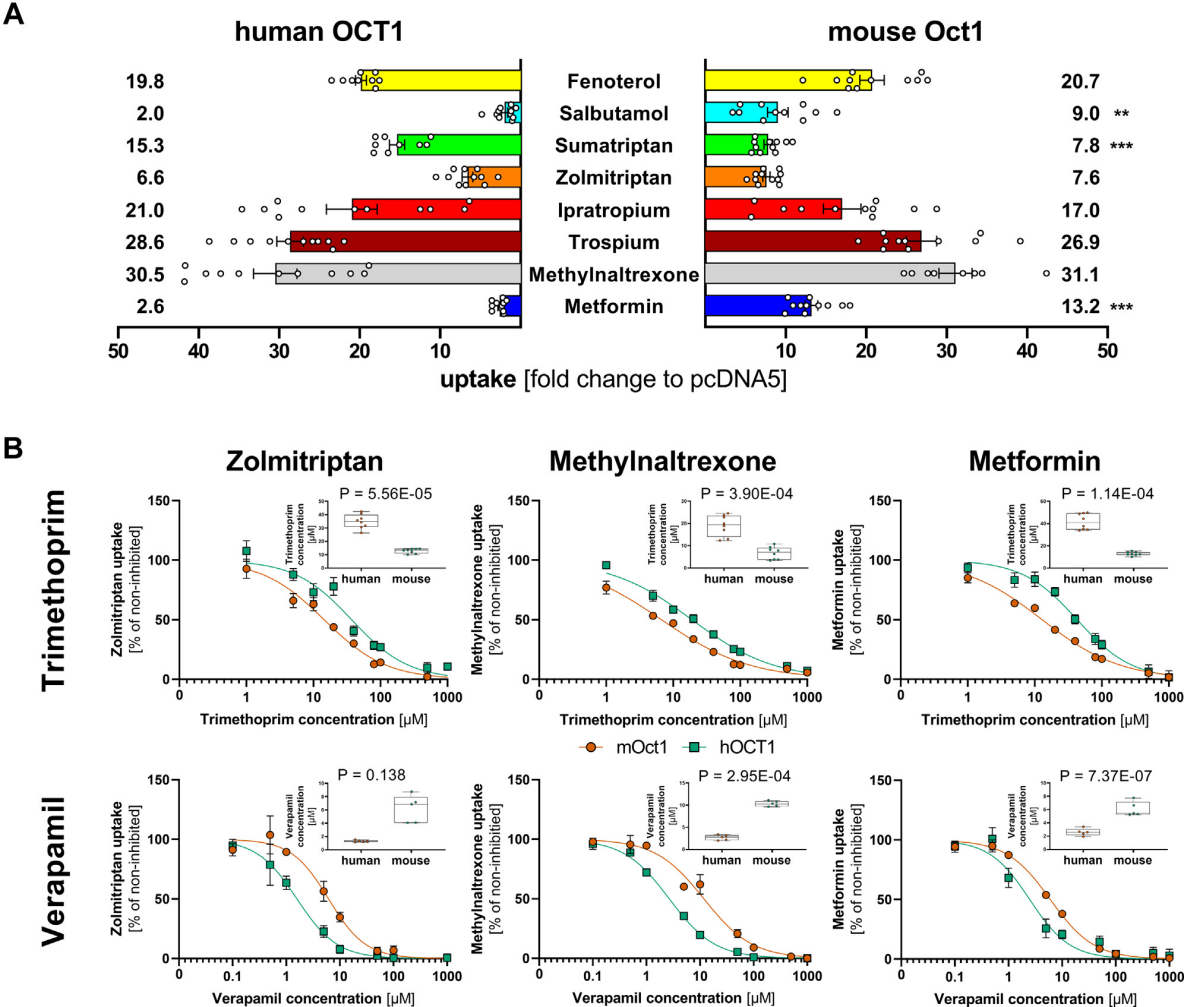
Differences in the uptake (A) and inhibition (B) of the cocktail between mouse and human OCT1. Comparison of the uptake of the cocktail between mouse and human OCT1 is shown as fold change to the negative control (A). *n* = 12 The respective substrates in the cocktail have the following colors: fenoterol yellow, salbutamol light blue, sumatriptan green, zolmitriptan orange, ipratropium red, trospium dark red, methylnaltrexone gray, and metformin dark blue. The inhibitory effects of trimethoprim and verapamil on mouse and human OCT1 uptake of the cocktail substrates (B). The inhibition curves of selected substrates (zolmitriptan, methylnaltrexone, and metformin) are shown and IC_50_ values for single experiments are shown as median and quantiles. Mouse Oct1 is shown in the blue green squares and human OCT1 in the vermillion circles. Significance was calculated using Mann–Whitney U test. *n* = 5–8 independent experiments.

Next, we analyzed species differences in the uptake and the inhibitory potencies. We used the cocktail to compare human and mouse OCT1 (Fig. 7). The uptake of metformin was 5.1-fold and of salbutamol was 4.5-fold stronger in mouse than in human OCT1 (Fig. 7A). In contrast, the uptake of sumatriptan was 2-fold stronger in human than in mouse OCT1.

We also observed strong differences in the inhibitory potency between the species. Trimethoprim inhibited more potently mouse than human OCT1. It inhibited metformin uptake with an IC_50_ of 12.9 *μ*M in mouse and 41.7 *μ*M in human OCT1 (*P* = .000114, Fig. 7B). Significant differences were observed also for inhibition of methylnaltrexone and zolmitriptan uptake (Fig. 7B and Table 4). However, the observed species differences in the inhibitory potencies were substrate-dependent, with sumatriptan showing smaller and ipratropium and trospium no species differences in trimethoprim inhibition (Table 4; Supplemental Fig. 1).

**Table 4:** Differences in the half-maximal inhibitory concentration (IC_50_ in *μ*M; mean ± SD) between human and mouse OCT1

		Inhibitors					
		Trimethoprim		Verapamil		Quinidine	
		*Human*	*Mouse*	*Human*	*Mouse*	*Human*	*Mouse*
Substrates	Fenoterol	45.0 ± 3.45	23.3∗∗∗ ± 4.36	4.27 ± 0.63	12.6∗ ± 0.95	16.3 ± 1.83	10.4∗∗∗ ± 1.62
	Salbutamol	37.6 ± 7.77	11.3∗∗∗ ± 2.71	1.48 ± 0.18	6.91 ± 0.96	6.22 ± 1.48	8.95∗∗ ± 1.50
	Sumatriptan	12.0 ± 2.88	19.5∗ ± 3.49	8.40 ± 0.82	5.63∗∗∗ ± 1.38	25.2 ± 2.62	6.42 ± 1.19
	Zolmitriptan	34.9 ± 4.97	12.9∗∗∗ ± 1.64	1.30 ± 0.13	6.17∗∗∗ ± 1.82	8.61 ± 0.71	4.68 ± 0.40
	Ipratropium	10.9 ± 1.76	8.79 ± 1.22	8.66 ± 1.95	20.7∗ ± 3.46	28.9 ± 3.95	9.77∗∗ ± 0.57
	Trospium	18.5 ± 2.48	23.0 ± 3.76	5.99 ± 0.48	13.8 ± 1.56	18.1 ± 4.88	12.1∗∗∗ ± 2.08
	Methylnaltrexone	19.0 ± 4.36	6.70∗∗∗ ± 2.62	2.69 ± 0.52	10.3 ± 0.52	8.67 ± 1.48	4.37∗∗∗ ± 1.65
	Metformin	41.7 ± 6.65	12.9∗∗∗ ± 1.55	2.61 ± 0.48	6.06 ± 0.98	11.2 ± 2.48	7.02∗ ± 3.06

OCT, organic cation transporter.

∗*P* < .05, ∗∗ *P* < .01, ∗∗∗ *P* < .001 compared with human OCT1, Student’s *t* test with Bonferroni correction for 24 multiple tests.

In contrast, verapamil inhibited more potently human than mouse OCT1 (Fig. 7B). Also in this case, the extent of the species differences was dependent on the substrate. Strong differences in the inhibitory potency were observed when zolmitriptan, methylnaltrexone, and metformin were used as substrates, and no differences were observed when sumatriptan was used as the substrate (Table 4).

Interestingly, there were no strong species differences in quinidine inhibition (Supplemental Fig. 2). However, sumatriptan, which showed only limited species differences with the other inhibitors, showed the strongest differences between mouse and human OCT1.

## 4 Discussion

In this study, we established a substrate cocktail and used it to reveal substrate-specific differences in OCT1 inhibition as well as differences in substrate selectivity between OCT1 orthologs and paralogs. Indeed, we observed more than 6-fold differences in the inhibitory potency of the same perpetrator drug dependent on the victim drug used (Figs. 3 and 4; Table 3).

The substantial substrate-specific differences in inhibitory potencies have several implications. First, the choice of a victim drug may play a key role when evaluating the inhibitory potencies in line with the FDA and ITC recommendations. Our data suggest that regardless of the victim drug used, the inhibitor trimethoprim should require follow-up in in vivo studies (Table 5). Indeed, previous studies demonstrated the potential of trimethoprim to inhibit OCT1 in humans (Vora et al, 2023) The authors showed that trimethoprim inhibited the OCT1-mediated uptake of thiamine (vitamin B1) with an IC_50_ of 4.5 *μ*M in vitro and this reflected in a measurable increase in thiamine plasma concentrations in humans after coadministration with trimethoprim. Another study reported that metformin AUC increased by 37% after the coadministration of trimethoprim (Grün et al, 2013). This is in line with our data, which suggest that trimethoprim may reach concentrations in the portal vein sufficient to inhibit OCT1-mediated uptake of all victim drugs tested, resulting in an increase in AUC ranging from 19% for fenoterol up to 79% for ipratropium.

**Table 5:** Analysis of potential DDIs in accordance with the Food and Drug Administration recommendations. Critical interactions exceeding the cutoff of R ≥ 1.1 are highlighted in bold

		Perpetrator							
		Fenoterol	Sumatriptan	Ipratropium	Trospium	Metformin	Quinidine	Verapamil	Trimethoprim
Victim drug	Fenoterol		1.00	1.00	1.00	1.00	**1.17**	**1.18**	**1.19**
	Salbutamol	1.00	1.00	1.00	1.01	1.00	**1.45**	**1.53**	**1.23**
	Sumatriptan	1.00		1.00	1.00	1.01	**1.11**	1.09	**1.71**
	Zolmitriptan	1.00	1.00	1.00	1.01	1.00	**1.32**	**1.60**	**1.24**
	Ipratropium	1.00	1.01		1.01	1.01	**1.10**	1.09	**1.79**
	Trospium	1.00	1.01	1.00		1.00	**1.15**	**1.13**	**1.46**
	Methylnaltrexone	1.00	1.00	1.00	1.01	1.01	**1.32**	**1.29**	**1.45**
	Metformin	1.00	1.00	1.00	1.01		**1.25**	**1.30**	**1.20**

DDI, drug-drug interaction; R, drug drug interaction risk.

In contrast, the recommendation for verapamil may vary depending on the victim drug used (Table 5). If sumatriptan or ipratropium are used as victim drugs, there is a risk of underestimating potential DDIs. This is not the case when zolmitriptan or methylnaltrexone are used as victim drugs. Importantly, our data also suggest that there is no single “perfect” victim drug that can consistently predict DDIs accurately. For example, although sumatriptan and ipratropium perform well with trimethoprim as an inhibitor, their performance is less reliable when verapamil is used as an inhibitor.

Regulatory guidelines suggest metformin or metformin and TEA^+^ to be used as victim drug for assessing potential DDIs at OCT2 in vivo or in vitro, respectively (ICH, 2024). Currently, neither the EMA nor the ITC provide a clear recommendation as to which substrate should actually be used. In our case, we have developed a validated cocktail with 8 substrates. Using this cocktail may have advantages in identifying potential DDIs at OCT1 and help prevent underestimation while requiring the same number of in vitro experiments as analyzing a single victim drug.

We identified 2 groups of inhibitors based on their inhibitory potency profiles: fenoterol, verapamil, quinidine, and partially ipratropium on one hand and sumatriptan, trimethoprim, and metformin on the other hand. The inhibition profiles resemble a lock-and-key match (Figs. 3 and 4), suggesting that these 2 groups of substrates and inhibitors may not have overlapping binding positions in the OCT1 substrate-binding pocket. This could not be claimed for trospium, which has an inhibition profile with only a weak correlation with those of ipratropium and nonsignificant with the rest of the group.

The available cryogenic electron microscopy data cannot explain the existence of 2 groups of inhibitors. None of the resolved OCT1 structures suggest the presence of 2 independent substrate-binding sites (Suo et al, 2023; Zeng et al, 2023; Zhang et al, 2024). OCT1 structures have also been resolved with some of the substrates (eg, metformin and fenoterol) and some of the inhibitors (eg, verapamil) used in this study. No significant differences were observed in the amino acids proposed by the authors to interact with inhibitors from the first group (verapamil and fenoterol) and those from the second group (metformin). However, only a limited number of conformations have been resolved, making it difficult to draw more precise conclusions about the OCT1 structures potentially involved in specific interactions with both groups of inhibitors.

We observed substantial differences in the substrate spectrum between OCT1 and its closely related paralogs OCT2 and OCT3 (Fig. 6). This is consistent with previous reports (Gebauer et al, 2021, 2022). On one hand, these differences are important for estimating the contribution of the different paralogs to the pharmacokinetics in humans. On the other hand, analyzing these differences in detail has the potential to help uncover the mechanisms underlying the broad substrate specificity of these transporters, a strategy that we previously used to study the differences between human and mouse orthologs (Meyer et al, 2022).

The cocktail could be used with only marginal adjustments to assess DDIs at other OCTs with a broad substrate spectrum like the MATEs. MATE1 and MATE2K transported almost all substrates in the cocktail, except for salbutamol (Fig. 6). Therefore, the same cocktail, excluding salbutamol, could be directly used to analyze substrate-specific differences in DDIs involving MATEs. This strategy can also be extended to other transporter groups, such as OATs, OATPs, or even promiscuous efflux transporters like MDR1. In these cases, a new selection of multiple victim drugs of interest would be required.

We also observed species-specific differences in the inhibitory potencies between human and mouse OCT1 (Fig. 7 and Table 4), which may have implications regarding the translatability of data from mouse models to humans during drug development (Supplemental Table 6). All 3 inhibitors—quinidine, verapamil, and trimethoprim—showed species-specific differences in their inhibitory potencies. Although quinidine and trimethoprim were more potent inhibitors of mouse OCT1, verapamil inhibited more potently human OCT1 for most tested substrates (Table 4). Using mouse OCT1 with single victim drugs such as methylnaltrexone, trospium, ipratropium, or fenoterol could lead to an underestimation of verapamil’s potential to cause DDIs in humans (R < 1.1, Supplemental Table 6). However, when applying a cocktail of multiple substrates, multiple inhibition signals would be detected even when using only mouse OCT1 (eg, sumatriptan, zolmitriptan, salbutamol, or metformin, Supplemental Table 6) warranting further investigations.

This cocktail was designed to cover a broad spectrum of structures to identify all potential DDIs at OCT1, as well as differences in uptake between orthologs or paralogs. Once such differences are identified, the same in vitro cocktail strategy can be used to pinpoint the specific ligand structural moieties responsible for these differences. For example, if differences in uptake between ipratropium and trospium via OCT3 are observed (Fig. 6), a cocktail of 6 to 7 tropane-based anticholinergic drugs can be developed to further narrow down the structural features causing these transport differences. Similar cocktails with a narrower structural spectrum can be developed for biguanides, beta-2 adrenergic drugs, triptans, and tetraalkylammonium compounds. This approach allows the identification of critical ligand structural moieties with only a handful of experiments, significantly enhancing the efficiency of substrate-structure analysis. Another promising application could be using the cocktail approach to study commonly coadministered drug combinations in clinical practice.

The in vitro cocktail approach has some limitations. It enables assessing DDIs or ortholog and paralog substrate specificity only at 1 concentration of the substrate, eg, victim drug. This allows determining only IC_50_ but not K_i_ as inhibition parameter. However, all concentrations were chosen to be far below the K_M_ of each substrate and thus correspond better to the commonly observed plasma concentrations of the victim drug in vivo.

In conclusion, we demonstrated that the inhibitory potency of an OCT1 perpetrator drug could vary significantly depending on the victim drug used. Based on this finding, we strongly recommend testing for potential DDIs at OCT1 using more than 1 victim drug. We also present a validated cocktail that allows for an efficient initial screening of potential DDIs at OCT1 in vitro with an effort comparable to testing a single substrate. This approach may be relevant and extendable to other transporters with broad substrate specificity, such as OATPs or P-gp.

## Conflict of interest

The authors declare no conflicts of interest.
