# Supplementary material for: Substrate-specific inhibition of organic cation transporter 1 revealed using a multisubstrate drug cocktail
Source: Drug Metab Dispos. 2025 Mar 28;53(5):100074. doi: 10.1016/j.dmd.2025.100074 (PMC12163485; doi:10.1016/j.dmd.2025.100074)
Supplement: Supplementary Material 1 [file mmc1.docx]

**Supplemental Material**

**Substrate-specific inhibition of organic cation transporter 1 revealed using a multi-substrate drug cocktail**

Vincent Rönnpagel, Felix Morof, Sarah Römer, Marleen J. Meyer-Tönnies, Mladen V. Tzvetkov*

Department of General Pharmacology, Institute of Pharmacology, C_DAT, University Medicine Greifswald (V.R., F.M., S.R., M.J.M., M.V.T.)

**Supplemental Table 1.** Precision and accuracy of intra-day, inter-day, and reinjection comparison

| **Analyte** | **Nominal concentration [µM]** | **Intra-day**  **[%, n = 5]** | | **Inter-day**  **[%, n = 15]** | | **Reinjection**  **[%, n = 5]** | |
| --- | --- | --- | --- | --- | --- | --- | --- |
|  |  | **Precision** | **Accuracy** | **Precision** | **Accuracy** | **Precision** | **Accuracy** |
| **Fenoterol** | 0.0004 | 1.21 - 9.15 | -12.7 - -3.9 | 7.30 | -7.75 | 8.24 | -1.90 |
|  | 0.0012 | 2 - 9.67 | -5.17 - 1.67 | 7.38 | -2.19 | 2.33 | 2.83 |
|  | 0.16 | 3.08 - 8.51 | -5.5 - 1.5 | 6.42 | -2.71 | 1.80 | -0.75 |
|  | 0.3 | 8.47 - 8.95 | -4.27 - -1 | 8.24 | -3.16 | 3.05 | -2.87 |
| **Salbutamol** | 0.0001 | 2.34 - 6.29 | 1.12 – 3.54 | 3.99 | 2.20 | 1.95 | 6.60 |
|  | 0.0003 | 3.71 - 6.75 | -0.07 - 3.73 | 4.95 | 2.42 | 9.60 | -2.67 |
|  | 0.04 | 2.97 - 7.12 | -12.55 - -3.80 | 6.67 | -9.43 | 5.28 | -2.50 |
|  | 0.075 | 3.19 - 4.69 | -11.73 - 1.36 | 7.14 | -5.83 | 6.89 | 0.64 |
|  |  |  |  |  |  |  |  |
| **Sumatriptan** | 0.0004 | 1.64 – 9 | -0.95 - 5.15 | 7.00 | 1.92 | 1.11 | 4.85 |
|  | 0.0012 | 2.98 - 7.4 | -5 - 1.5 | 5.88 | -1.83 | 4.32 | 2.33 |
|  | 0.16 | 5.49 - 7.11 | -8 - 1.25 | 7.26 | -3.00 | 5.25 | -1.62 |
|  | 0.3 | 4.64 - 5.55 | -6.8 - 0 | 5.62 | -2.56 | 5.34 | 0.73 |
| **Zolmitriptan** | 0.0004 | 2.85 - 5.43 | -9.50 - -3.8 | 4.70 | -6.03 | 0.95 | 2.80 |
|  | 0.0012 | 1.66 - 8.13 | -9.40 - -2.5 | 6.71 | -6.91 | 2.50 | 4.50 |
|  | 0.16 | 4.72 - 6.97 | -9.5 - -4.75 | 5.72 | -7.63 | 8.12 | -10.50 |
|  | 0.3 | 1.33 - 6.14 | -3.47 - 0.33 | 4.48 | -1.67 | 4.79 | -9.67 |
| **Ipratropium** | 0.0004 | 3.16 - 7.63 | -8.20 - 1.2 | 7.26 | -4.30 | 7.72 | -4.10 |
|  | 0.0012 | 5.32 - 7.60 | -8.50 - -3.67 | 6.72 | -6.39 | 5.65 | -3.00 |
|  | 0.16 | 2.9 - 7.18 | -7.88 - 4.5 | 7.73 | -0.46 | 3.68 | 1.50 |
|  | 0.3 | 3.81 - 6.69 | -9.67 - 4.13 | 7.86 | -1.91 | 3.53 | 0.20 |
| **Trospium** | 0.0001 | 4.42 - 6.65 | -2.46 - 8.8 | 6.86 | 2.78 | 5.56 | -9.02 |
|  | 0.0003 | 7.52 - 9.58 | -3.13 - 0.33 | 8.55 | -1.47 | 6.80 | -8.80 |
|  | 0.04 | 4.14 - 5.18 | -9.55 - -4.05 | 5.02 | -6.53 | 7.36 | -2.50 |
|  | 0.075 | 2.67 - 4.49 | -9.28 - 1.17 | 5.75 | -4.66 | 2.25 | 2.91 |
| **Methylnaltrexone** | 0.0004 | 1.52 - 8.26 | 1.95 - 3.4 | 5.50 | 2.80 | 9.43 | -7.45 |
|  | 0.0012 | 2.71 - 7.47 | -6.67 - -3.83 | 5.64 | -5.17 | 8.65 | -5.83 |
|  | 0.16 | 2.48 - 4.43 | -3.25 - 1.63 | 4.10 | -1.04 | 0.68 | 0.13 |
|  | 0.3 | 2.78 - 5.60 | -1.4 - 5.93 | 4.92 | 1.64 | 4.19 | -2.13 |
| **Metformin** | 0.004 | 5.66 - 8.28 | -9 - -0.15 | 8.14 | -3.18 | 4.90 | 1.82 |
|  | 0.012 | 6.75 - 9.87 | 0.17 - 2.83 | 7.70 | 1.50 | 3.30 | 0.17 |
|  | 1.6 | 2.48 - 4.67 | 7.25 - 9.87 | 4.02 | 8.33 | 4.65 | -0.50 |
|  | 3.0 | 1.83 - 6.46 | 1.8 - 4.87 | 4.04 | 3.18 | 3.48 | 4.33 |

**Supplemental Table 2.** Freeze-thaw, rack, and bench top stability

| **Analyte** | **Nominal concentration [µM]** | **Freeze-thaw stability**  **[%; n = 3]** | | **Rack stability**  **[%; n = 5]** | | **Bench top stability**  **[%; n = 5]** | |
| --- | --- | --- | --- | --- | --- | --- | --- |
|  |  | **Precision** | **Accuracy** | **Precision** | **Accuracy** | **Precision** | **Accuracy** |
| **Fenoterol** | 0.0012 | 4.39 | 2.22 | 6.99 | -7.22 | 9.81 | 7.88 |
|  | 0.3 | 0.51 | 2.78 | 6.41 | 6.53 | 4.00 | -0.87 |
| **Salbutamol** | 0.0003 | 9.09 | -5.22 | 3.92 | 4.07 | 2.64 | 6.93 |
|  | 0.075 | 9.27 | -5.42 | 4.91 | 6.59 | 6.67 | -4.11 |
| **Sumatriptan** | 0.0012 | 5.34 | -1.67 | 9.27 | -4.82 | 8.82 | -4.17 |
|  | 0.3 | 8.13 | 0 | 2.21 | -3.87 | 8.13 | 1.67 |
| **Zolmitriptan** | 0.0012 | 5.84 | 8.03 | 2.64 | -5.73 | 8.31 | 5.67 |
|  | 0.3 | 7.80 | -3.22 | 5.09 | -9.87 | 3.70 | 1.20 |
|  |  |  |  |  |  |  |  |
| **Ipratropium** | 0.0012 | 5.19 | 1.39 | 2.86 | 1.50 | 9.58 | -0.33 |
|  | 0.3 | 2.85 | 9.22 | 5.02 | 0.40 | 2.65 | -5.80 |
| **Trospium** | 0.0003 | 5.98 | 6.00 | 4.37 | -7.13 | 5.05 | 6.73 |
|  | 0.075 | 0.88 | -2.93 | 3.13 | -6.56 | 4.98 | 3.55 |
| **Methylnaltrexone** | 0.0012 | 6.67 | -5.56 | 5.90 | 5.17 | 7.54 | 5.33 |
|  | 0.3 | 5.82 | 3.22 | 3.22 | 0.87 | 1.46 | 7.07 |
| **Metformin** | 0.012 | 5.30 | -5.56 | 5.56 | 4.86 | 4.93 | 4.38 |
|  | 3 | 3.39 | -3.56 | 2.17 | 0.40 | -5.83 | -4.13 |

**Supplemental Table 3.** Linear ranges, linear equations, and correlation coefficients of the eight cocktail substrates

| Analyte | Linear range [µM] | Regression equation | coefficient of  determination |
| --- | --- | --- | --- |
| Fenoterol | 0.0004 - 0.3 | y = 46.1x + 0.00558 | 0.9995 ± 0.00021 |
| Salbutamol | 0.0001 - 0.075 | y = 31.7x – 0.00110 | 0.9993 ± 0.00042 |
| Sumatriptan | 0.0004 - 0.3 | y = 23.9x – 0.00029 | 0.9998 ± 0.00006 |
| Zolmitriptan | 0.0004 - 0.3 | y = 9.34x + 0.00006 | 0.9997 ± 0.00005 |
| Ipratropium | 0.0004 - 0.3 | y = 152x + 0.00412 | 0.9999 ± 0.00003 |
| Trospium | 0.0001 - 0.075 | y = 349x + 0.00712 | 0.9998 ± 0.00004 |
| Methylnaltrexone | 0.0004 - 0.3 | y = 5.09x – 0.00033 | 0.9991 ± 0.00031 |
| Metformin | 0.0004 - 0.3 | y = 4.78x + 0.00392 | 0.9991 ± 0.00031 |

**Supplemental Table 4**. Pharmacokinetic parameters used for estimating the potential for DDIs

|  | Ipratropium | Fenoterol | Sumatriptan | Trospium | Metformin | Trimethoprim | Verapamil | Quinidine |
| --- | --- | --- | --- | --- | --- | --- | --- | --- |
| Administration | inhalation | i.v. | p.o. | p.o. | p.o. | p.o. | p.o. | p.o. |
| Dose [mg] | 0.08 | 0.18 | 100 | 30 | 500 | 160 | 120 | 400 |
| MW [g/mol] | 412.36 | 303.36 | 295.40 | 392.52 | 129.17 | 290.32 | 454.61 | 324.42 |
| c_max_ = I_max_ [ng/mL] | 0.08 | 1.23 | 54 | 5.20 | 1600 | 1200 | 272 | 1300 |
| f_u_ [%] | 0.91 | 0.53 | 0.79 | 0.33 | 1.00 | 0.63 | 0.10 | 0.13 |
| t_1/2_ [h] | n/a | 1.27 | 1.00 | 9.9 | 1.74 | 10.00 | 4.00 | 6.20 |
| t_max_ [h] | n/a | n/a | 1.50 | 6.7 | 1.90 | 2.00 | 1.10 | 2.00 |
| k_e_ [h^-1^] | n/a | n/a | 0.69 | 0.07 | 0.398 | 0.069 | 0.173 | 0.112 |
| k_a_ [h^-1^] | n/a | n/a | 0.64 | 0.273 | 0.679 | 1.66 | 2.65 | 1.36 |
| I_max, portal_ [ng/mL] | 0.08 | 1.23 | 714.8 | 89.63 | 5100 | 3932 | 3550 | 6908 |
| I _max, portal, u_ [µM] | **0.0002** | **0.0021** | **1.91** | **0.08** | **39.48** | **8.53** | **0.78** | **2.77** |

*n/a, not applicable*

**Supplemental Table 5**. Substrate specific K_M_ and v_max_ values for hOCT1 tested alone or in combination with the cocktail (mean ± SD)

| Substrate | alone | | in cocktail | |
| --- | --- | --- | --- | --- |
|  | K_M_  [µM] | v_max_ [pmol x min^-1^ x mg protein^-1^] | K_M_  [µM] | v_max_ [pmol x min^-1^ x mg protein^-1^] |
| Fenoterol | 0.94 ± 0.24 | 44.4  ± 10.5 | 0.95  ± 0.38 | 43.9  ± 9.66 |
| Sumatriptan | 99.9  ±13.2 | 1887  ± 556 | 131  ± 24.1 | 1844  ± 533 |
| Trospium | 16.9  ± 2.46 | 647  ± 207 | 13.9  ± 1.61 | 637  ± 227 |
| Ipratropium | 10.6  ± 3.54 | 924  ± 224 | 11.7  ± 3.3 | 952  ± 134 |
| Metformin | 1381  ± 512 | 6999  ± 2065 | 1999  ± 338 | 5251  ± 1311 |

**Supplemental Table 6**. Comparison of the predicted risk of drug-drug interactions (expressed as R; for details, see the main text) estimated using in vitro inhibitory experiments with mouse and human OCT1. The R-values indicating potential DDI risk are highlighted in pink. Also shown are the differences (Δ) in R between mouse and human OCT1. The scale for ΔR ranges from red (mouse measurements underestimate the risk) to green (mouse measurements overestimate the risk).

|  | | **Perpetrator** | | | | | | | | |
| --- | --- | --- | --- | --- | --- | --- | --- | --- | --- | --- |
|  |  | **Quinidine** | | | **Verapamil** | | | **Trimethoprim** | | |
|  |  | **mouse** | **human** | **ΔR** | **mouse** | **human** | **ΔR** | **mouse** | **human** | **ΔR** |
| Victim drug | Fenoterol | 1.27 | 1.17 | 0.1 | 1.06 | 1.18 | -0.12 | 1.37 | 1.19 | 0.18 |
|  | Salbutamol | 1.31 | 1.45 | -0.14 | 1.11 | 1.53 | -0.42 | 1.76 | 1.23 | 0.53 |
|  | Sumatriptan | 1.43 | 1.11 | 0.32 | 1.14 | 1.09 | 0.05 | 1.44 | 1.71 | -0.27 |
|  | Zolmitriptan | 1.59 | 1.32 | 0.27 | 1.13 | 1.6 | -0.47 | 1.66 | 1.24 | 0.42 |
|  | Ipratropium | 1.28 | 1.1 | 0.18 | 1.04 | 1.09 | -0.05 | 1.97 | 1.79 | 0.18 |
|  | Trospium | 1.23 | 1.15 | 0.08 | 1.06 | 1.13 | -0.07 | 1.37 | 1.46 | -0.09 |
|  | Methylnaltrexone | 1.63 | 1.32 | 0.31 | 1.08 | 1.29 | -0.21 | 2.27 | 1.45 | 0.82 |
|  | Metformin | 1.39 | 1.25 | 0.14 | 1.13 | 1.3 | -0.17 | 1.66 | 1.2 | 0.46 |


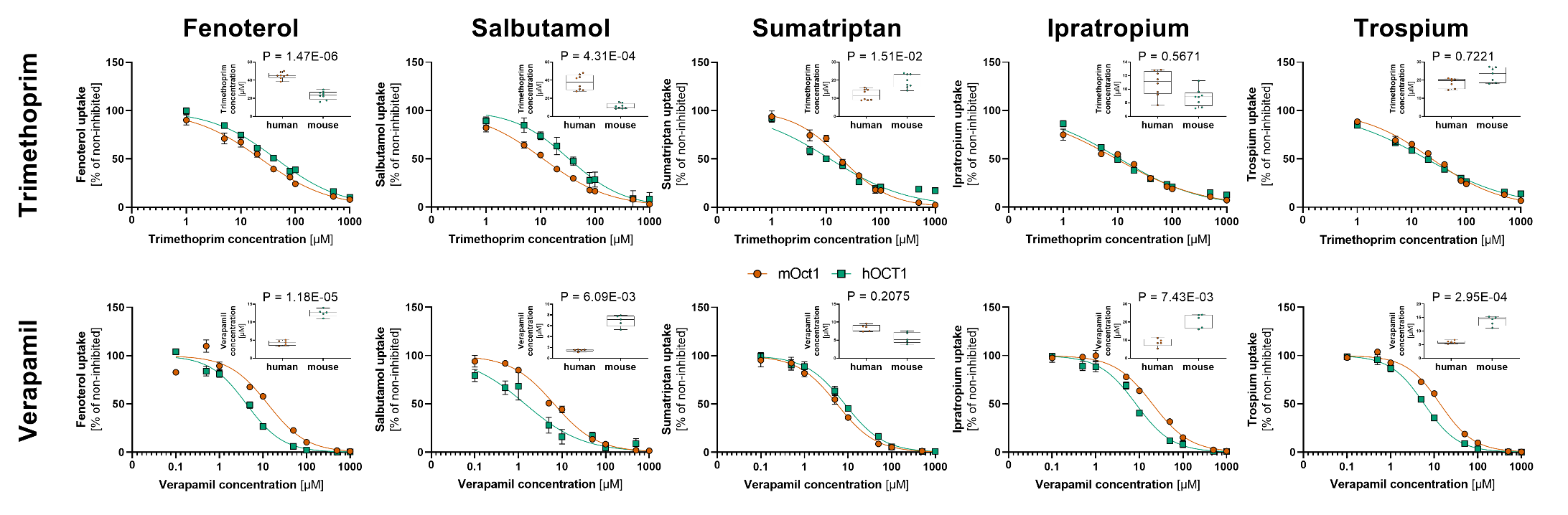


**Supplemental Figure 1**. **Differences in the inhibition of the cocktail between mouse and human OCT1 for trimethoprim and verapamil** Comparison of the inhibitory effects of trimethoprim and verapamil between mouse and human OCT1 on uptake of the cocktail. The inhibition curves are shown and IC_50_ values for single experiments are shown as median and quantiles. Mouse Oct1 is shown in the blue green squares and human OCT1 in the vermillion circles. Significance was calculated using t-test for independent samples with Bonferroni correction. n = 5-8 independent experiments


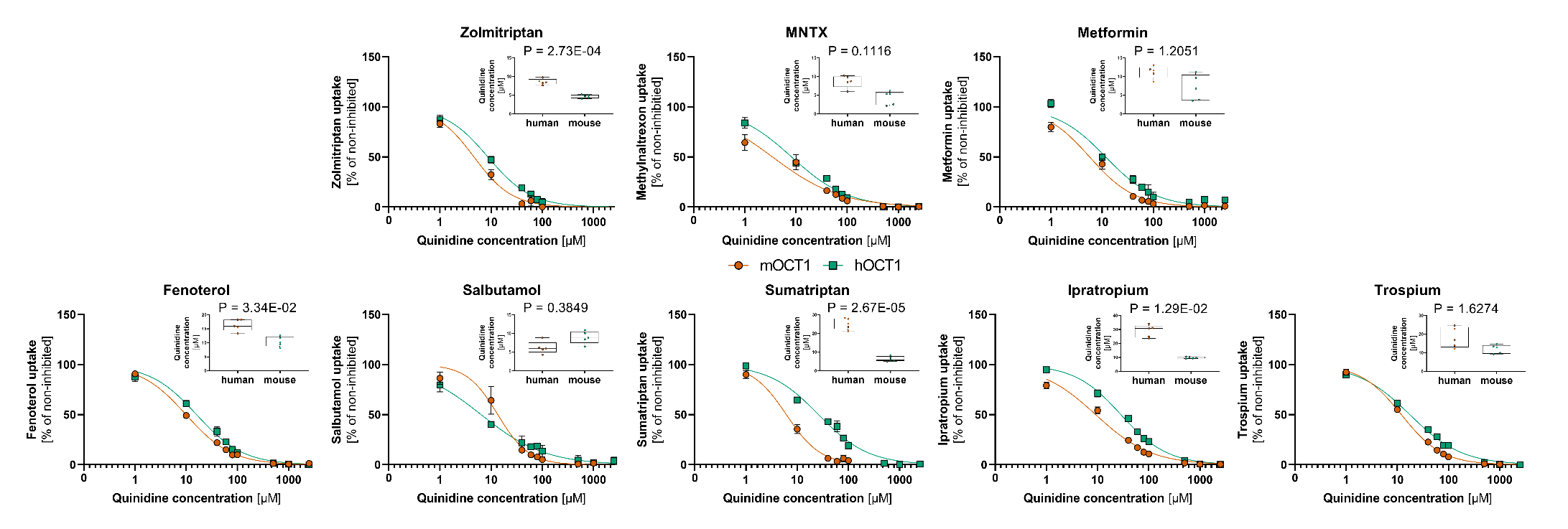


**Supplemental Figure 2. Differences in the inhibition of the cocktail between mouse and human OCT1 for quinidine** Comparison of the inhibitory effects of quinidine between mouse and human OCT1 on uptake of the cocktail. The inhibition curves were shown and IC_50_ values for single experiments are shown as median and quantiles. Mouse Oct1 is shown in the blue green squares and human OCT1 in the vermillion circles. Significance was calculated using t-test for independent samples with Bonferroni correction. n = 5 independent experiments

**Supplemental Methods**

**Preparation of stock solutions, standards and quality control samples**

The stock solutions of the internal standards and the analytes were prepared by dissolving them in water. Only zolmitriptan, trimethoprim, and verapamil were dissolved in DMSO. All dilutions of the stock solutions were prepared in water. Stock solutions were prepared at concentrations 10 mM for fenoterol, salbutamol, sumatriptan, ipratropium, trospium, methylnaltrexone, L-carnitine-d9, and quinidine and 100 mM for zolmitriptan, metformin, L-ergothioneine, trimethoprim, and verapamil. Working solutions were prepared at a concentration of 1 mM for each analyte except for salbutamol, metformin, trimethoprim, and quinidine, where the stock solution is also the work solution as well as verapamil where the working solution was obtained with a concentration of 20 mM. A concentration of 1 mg/ml was obtained for fenoterol-d6, sumatriptan-d6, trospium-d8 and isobutyryl-L-carnitine-d6 (IBC). The working solution for the internal standards was prepared at a concentration of 10 µg/ml. The internal standards were prepared with a second working solution of the analytes, with a concentration of 100 µM for fenoterol, sumatriptan, zolmitriptan, ipratropium, methylnaltrexone and L-carnitin-d9, 20 µM for salbutamol and trospium, and 1 mM for metformin and L-ergothioneine. These were then diluted with 80% acetonitrile containing the internal standards at a concentration of 10 ng/mL by serial dilution to obtain standard concentrations of 0.0001, 0.0002, 0.0005, 0.001, 0.004, 0.03 and 0.1 µM for salbutamol and trospium, of 0.0004, 0.0008, 0.002, 0.004, 0.016, 0.12 and 0.4 µM for fenoterol, sumatriptan, zolmitriptan, ipratropium, methylnaltrexone and L-carnitin-d9, and of 0.004, 0.008, 0.02, 0.04, 0.16, 1.2 and 4 µM for metformin and L-ergothioneine. The quality control (QC) samples were also prepared with the second working solution by serial dilution with 80% acetonitrile containing the internal standards at a concentration of 10 ng/mL to create the lowest level of quantitation QC (LLQC), QC low, QC medium and QC high at 0.0001, 0.0003, 0.04 and 0.075 µM for salbutamol and trospium, at 0.0004, 0.0012, 0.16 and 0.3 µM for fenoterol, sumatriptan, zolmitriptan, ipratropium and methylnaltrexone, and at 0.004, 0.012, 1.6 and 3 µM for metformin. Stock and working solutions of internal standards and analytes were stored at -20°C.

The quality control acceptance criteria for inter-day and intra-day accuracy and precision were a mean accuracy (percent relative error (%RE)) and precision (percent coefficient of variation (%CV); n=5) of ≤ 20 at LLQC and ≤ 15 at all other concentrations. The criteria for showing the stability (n=5) were mean %RE and %CV versus T_0_ of ≤ 15. The reinjection stability of the samples was assessed by reinjecting an accuracy and precision batch that had been analyzed two days prior and stored at 4°C in the autosampler (n=5).

The bench-top stability and rack stability of the analytes in 80% acetonitrile containing the internal standards was assessed for 24 h at room temperature and 4°C at the QC low and QC high concentrations. Freeze-thaw stability in 80% acetonitrile containing the internal standards was assessed over three freeze-thaw cycles at the QC low and QC high concentrations on consecutive days. QC samples were stored at -20°C. Aliquots were thawed at room temperature for a minimum of 1 h and then returned to storage for a minimum of 20 h for each cycle.

**Supplemental Results**

**Validation of the LC-MS/MS method**

The method was characterized with three batch runs to determine accuracy and precision and another four runs to complete the other quality control criteria.

Seven standard concentrations were measured at the beginning. A weighted (1/x) linear regression led to the best fit over the required concentration range (Table 3). The lower limit of quantification (LLOQ) was below 0.5 nM and the linear range was close to three degrees of magnitude for each of the substrates tested. The coefficient of determination (R^2^) was above 0.999 for each of the substrates (Table 3).

Accuracy and precision were assessed with three batch runs on three consecutive days. Each day had five sets of QC samples. The intra-day accuracy over these three days ranged from -12.7 to 9.87 and precision ranged from 1.21 to 9.9. Inter-day accuracy ranged from 3.99 to 8.55 and inter-day precision from -9.43 to 8.33. All substrates were stable over the four QC concentrations (LLOQ, low, medium and high) for at least 48h when kept at 4°C. The %RE ranged from 0.68 to 9.6 and %CV ranged from -10.5 to 6.6 (Supplemental Table 1).

All substrates demonstrated stability in 80% acetonitrile containing the internal standards when kept at 4°C after 48 h over the four QC concentrations (%RE ranged from 0.58 - 9.6; %CV ranged from -10.5 - 6.6)

All substrates (Table 2) demonstrated stability in 80% acetonitrile containing the internal standards at RT for 24 h at both QC concentrations (%RE of -5.83 - 6.93 (QC low) and -5.8 - 7.07 (QC high), relative to T_0_).

All substrates demonstrated stability in 80% acetonitrile containing the internal standards on 4°C for 48h at both QC concentrations (%RE of -7.22 - 5.17 (QC low) and -9.87 – 6.59 (QC high), relative to T_0_).

All substrates demonstrated stability in 80% acetonitrile containing the internal standards over three freeze-thaw cycles (-20°C to RT) at both QC concentrations (%RE of -5.56 - 8.03 (QC low) and -5.42 - 9.22 (QC high), relative to T_0_, Supplemental Table 2)
